# NMR-based Metabolomic Techniques Identify the Toxicity of Emodin in HepG2 Cells

**DOI:** 10.1038/s41598-018-27359-4

**Published:** 2018-06-20

**Authors:** Chang Chen, Jian Gao, Tie-Shan Wang, Cong Guo, Yu-Jing Yan, Chao-Yi Mao, Li-Wei Gu, Yang Yang, Zhong-Feng Li, An Liu

**Affiliations:** 1 Institute of Chinese Materia Medica, China Academy of Chinese Medical Sciences, Beijing, China; 2 Beijing University of Chinese Medicine, Beijing, China; 3 Department of Chemistry, Capital Normal University, Beijing, China; 4 China Academy of Chinese Medical Sciences, Beijing, China

## Abstract

Emodin is a natural anthraquinone derivative that is present in various herbal preparations. The pharmacological effects of emodin include anticancer, hepatoprotective, anti-inflammatory, antioxidant and even antimicrobial activities. However, emodin also has been reported to induce hepatotoxicity, nephrotoxicity, genotoxicity and reproductive toxicity. The mechanism of emodin’s adverse effects is complicated and currently not well understood. This study aimed to establish a cell metabonomic method to investigate the toxicity of emodin and explore its potential mechanism and relevant targets. In the present study, metabonomic profiles of cell extracts and cell culture media obtained using the ^1^H NMR technique were used to assess emodin toxicity in HepG2 cells. Multivariate statistical analyses such as partial least squares-discriminant analysis (PLS-DA) and orthogonal partial least squares-discriminant analysis (OPLS-DA) were used to characterize the metabolites that differed between the control and emodin groups. The results indicated that emodin resulted in differences in 33 metabolites, including acetate, arginine, aspartate, creatine, isoleucine, leucine and histidine in the cell extract samples and 23 metabolites, including alanine, formate, glutamate, succinate and isoleucine, in the cell culture media samples. Approximately 8 pathways associated with these metabolites were disrupted in the emodin groups. These results demonstrated the potential for using cell metabonomics approaches to clarify the toxicological effects of emodin, the underlying mechanisms and potential biomarkers. Our findings may help with the development of novel strategies to discover targets for drug toxicity, elucidate the changes in regulatory signal networks and explore its potential mechanism of action.

## Introduction

Valuable safety information in traditional Chinese medicine (TCM) comes directly from its clinical use. This information can be used as an important reference for TCM safety evaluations. Emodin, which is an active ingredient of Chinese medical herbs, such as *Rheum palmatum*^1^, *Polygonum multiflorum*^2^ and *Polygonum cuspidatum*^3^, has been used for over 2,000 years in eastern Asia and is still present in various herbal preparations^4^. Previous studies have reported multiple pharmacological benefits of emodin, such as anticancer, hepatoprotective, anti-inflammatory, antioxidant and antimicrobial activities^5^. Emodin may be an effective therapeutic agent for prophylaxis and for various diseases including constipation, asthma, atopic dermatitis, atherosclerosis, hepatopathy osteoarthritis, diabetes and its complications, Alzheimer’s disease and tumors^5^. However, toxic effects of emodin, such as nephrotoxicity, hepatotoxicity, genotoxicity and reproductive toxicity, have also been reported^6–10^. Emodin was found to induce apoptotic responses in human hepatocellular carcinoma cell (HCC) lines and HepG2^11^. Emodin produced reactive oxygen species (ROS) in these cells, which reduced the intracellular mitochondrial transmembrane potential (Δ*Ψm*). This activated caspase-9 and caspase-3, leading to DNA fragmentation and apoptosis. Some studies have reported that emodin has the potential to disrupt glutathione and fatty acid metabolism in human liver cells^12^.

Drug-induced cytotoxicity is related to cell metabolism^13^. As a systemic approach, metabonomics adopts a “top-down” strategy to holistically reflect the function of organisms including terminal symptoms of metabolic network and understanding systemic metabolic changes caused by interventions^14^. Combining novel models with molecular profiling technologies, metabonomics provides new insights into the molecular basis of toxicity and provides a rich source of biomarkers that are urgently needed in 21st century toxicology research^15^. The metabolic profile of a whole organism does not provide relevant information about specific cell types under different conditions, which is crucial for creating a more holistic understanding of cell functions and for drug development^16^. Cell cultures may provide an alternative for understanding the specific metabolism of drug candidates^17^. Metabolic analysis of cell cultures has many potential applications and advantages over currently used methods for cell testing^18^.

Nuclear magnetic resonance (NMR) is an easy and convenient research tool. NMR is a suitable analysis method for examining complex compositions in omics research, particularly in modern TCM research. Principal component analysis (PCA), partial least squares-discriminant analysis (PLS-DA) and orthogonal partial least squares-discriminant analysis (OPLS-DA) methods were applied to maximize the distinction between groups, focusing on differences in metabolic variations, and to assess the correlations between the observed NMR results.

In this study, a novel attempt was made to explore the possible mechanisms and relevant targets of emodin toxicity based on NMR non-targeted metabolomics. The objective was to establish a cell metabonomic method and identify biomarkers for investigating the toxic effects of emodin. This study may provide new ideas and methods for investigating and understanding the toxicity mechanism of TCM.

## Results

### Emodin inhibited viability and HepG2 cell proliferation

**Figure 1:**
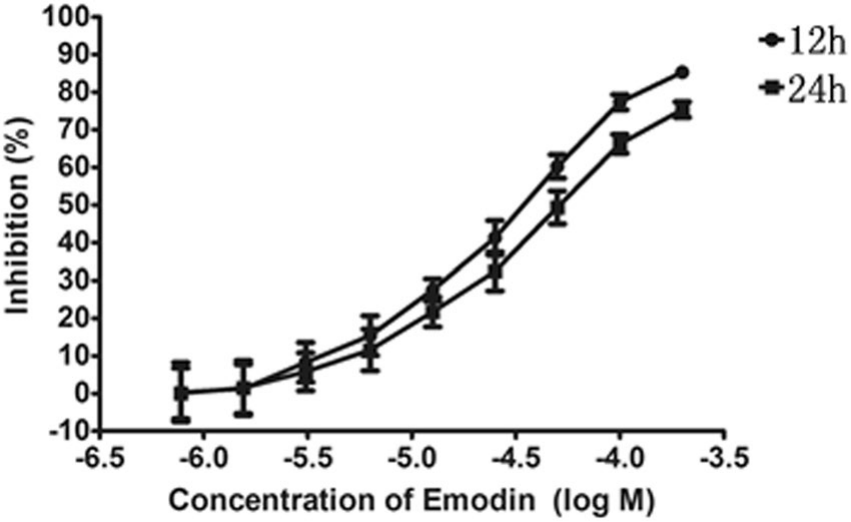
Emodin inhibited viability and proliferation in HepG2 cells. HepG2 cell were treated with different concentrations of emodin for 12 h and 24 h. Cell growth was determined using an MTT assay and was directly proportional to the absorbance at a wavelength of 570 nm. Data are expressed as the means ± S.D. for the three independent experiments.

An MTT assay showed that emodin inhibited HepG2 cell growth in both concentration- and time-dependent manners (Fig. 1). The 50% inhibitory concentration (IC50) values of emodin at 12 h and 24 h were 41.3 μM and 32.1 μM, respectively.

### Apoptosis caused by emodin

**Figure 2:**
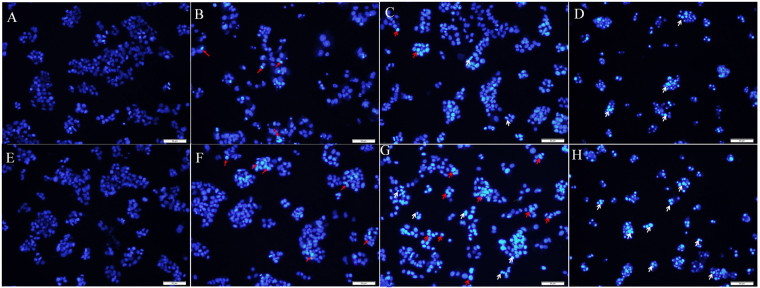
Fluorescent images of DAPI for the HepG2 cells exposed to 10, 50, or 100 μM of emodin for 12 h and 24 h. Different concentrations (10, 50, 100 μM) of emodin caused a decrease in cell numbers. The normal cells exhibited an evenly distributed, deep blue fluorescent color in the nucleus and the chromatin, while the apoptotic cells resulting from emodin exposure showed a bright white fluorescent color. In the emodin-treated groups, cells showed different degrees of chromatin condensation, nuclear condensation and fragmentation, as indicated by the arrows. The red arrows indicate chromatin condensation, while the white arrows indicate nucleus fragmentation. (**A**) Normal group (12 h); (**B**) low emodin group (10 μM, 12 h); (**C**) middle emodin group (50 μM, 12 h); (**D**) high emodin group (100 μΜ, 12 h); (**E**) normal group (24 h); (**F**) low emodin group (10 μM, 24 h); (**G**) middle emodin group (50 μM, 24 h); (**H**) high emodin group (100 μΜ, 24 h) (Bars = 50 μm).

After 12 h and 24 h of exposure, emodin was found to induce HepG2 cells apoptosis using a DAPI staining assay. As shown in Fig. 2, viable normal cells exhibited an evenly distribution and deep blue fluorescence in both the nucleus and chromatin, while the apoptotic cells showed bright white fluorescence. Changes in the DAPI staining were observed in apoptotic cells in the emodin-treated group. All of the emodin concentrations tested (10, 50, and 100 μM) decreased the number of cells. In the emodin-treated groups, the cells were found to have different degrees of chromatin condensation, nuclear condensation and fragmentation, as indicated by the arrows in Fig. 2. Increasing the incubation time produced fragmented nuclei at 24 h. The apoptotic area in the 100 μM emodin sample was obviously larger than that of the 50 μM and 10 μM samples. Exposure for 24 h and at 100 μM were chosen for conducting the following metabolomic study.

### ^1^H NMR analysis of cell extracts and culture media

**Figure 3:**
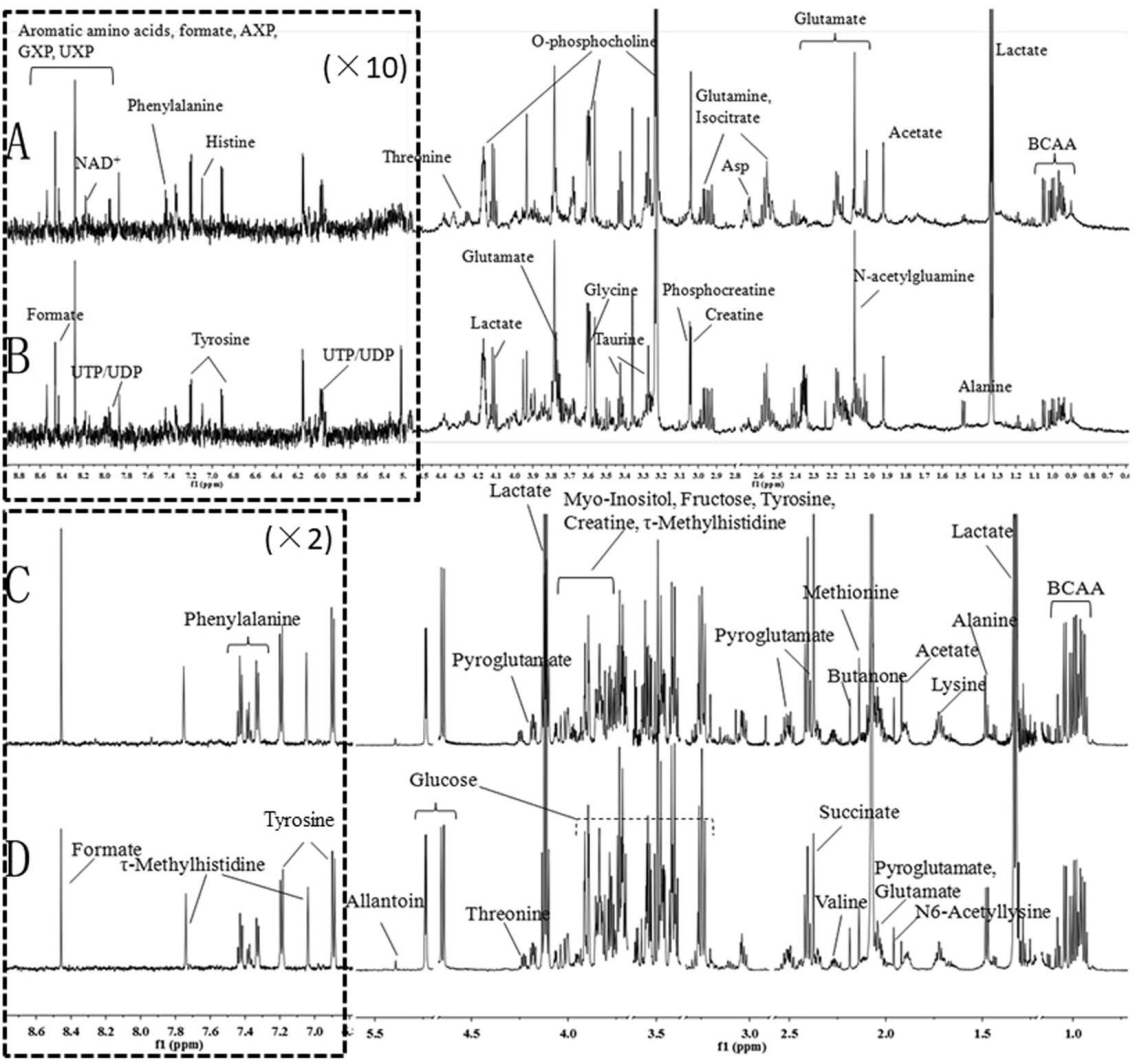
Representative one-dimensional 600 MHz ^1^H NMR spectra of cell extracts and culture media from the normal and high emodin groups. (**A**) spectra of cell extracts from the high emodin group; (**B**) spectra of cell extracts from the normal group; (**C**) Spectra of cell culture media from the high emodin group; (**D**) spectra of cell culture media from the normal group. BCAA (Branched-chain amino acid); AXP (AMP, ADP, ATP); GXP (GMP, GDP; GTP); UXP (UMP, UDP, UTP).

We analyzed cell extracts and culture media to investigate the potential for using metabolic fingerprinting to characterize emodin toxicological responses in liver cells. Figure 3 shows the 600 MHz ^1^H-NMR CPMG spectra of cell extracts and cell culture media from the high emodin group and control group. As used in previous studies^19–21^ and in our in-house NMR database (HMDB), the resonance assignments were performed and confirmed by a series of 2D NMR spectra including ^1^H-^1^H COSY, ^1^H-^13^C HMBC and ^1^H-^13^C HSQC. The cell extract NMR spectra were dominated by alanine (δ1.48, δ3.78), acetate (δ1.92), glutamine (δ2.13, δ2.44), leucine (δ0.96, δ1.69, δ3.74), valine (δ0.99, δ1.05, δ2.28), lactate (δ1.33, δ4.12), glucose (δ3.49, δ3.72, δ5.24), and glycine (δ3.58), among others. In the ^1^H NMR spectra of cell culture media, the primary changes were in signals for low-molecular-weight metabolites, such as threonine (δ1.33, δ3.56), isoleucine (δ1.02, δ1.46, δ8.09), valine (δ0.99, δ1.05, δ2.28), alanine (δ1.48, δ3.78), and glutamine (δ2.13, δ2.44). Multivariate data analysis was further performed to obtain a more detailed analysis of the metabolic differences between groups.

### Multivariate data analysis and the selection of potential biomarkers

PCA was performed using a mean-centered scaling approach. The data were visualized in the form of the principal component (PC) score plots to identify general metabolic trends and possible outliers. PCA from NMR data from HeLa cells and cell culture media revealed a clear dose-response (Figure S1) for emodin. The first two principal components (PC1 and PC2) explained 47.2% and 26.3% of the variation in the model in high-dose cell samples and 64.5% and 31.0% for the model and high-dose cell culture media samples. The majority of the samples were located within the 95% confidence interval. Further analyses of the NMR data from cells and cell culture media using PLS-DA showed a clear differentiation between the emodin group and control group (Figure S2).

**Figure 4:**
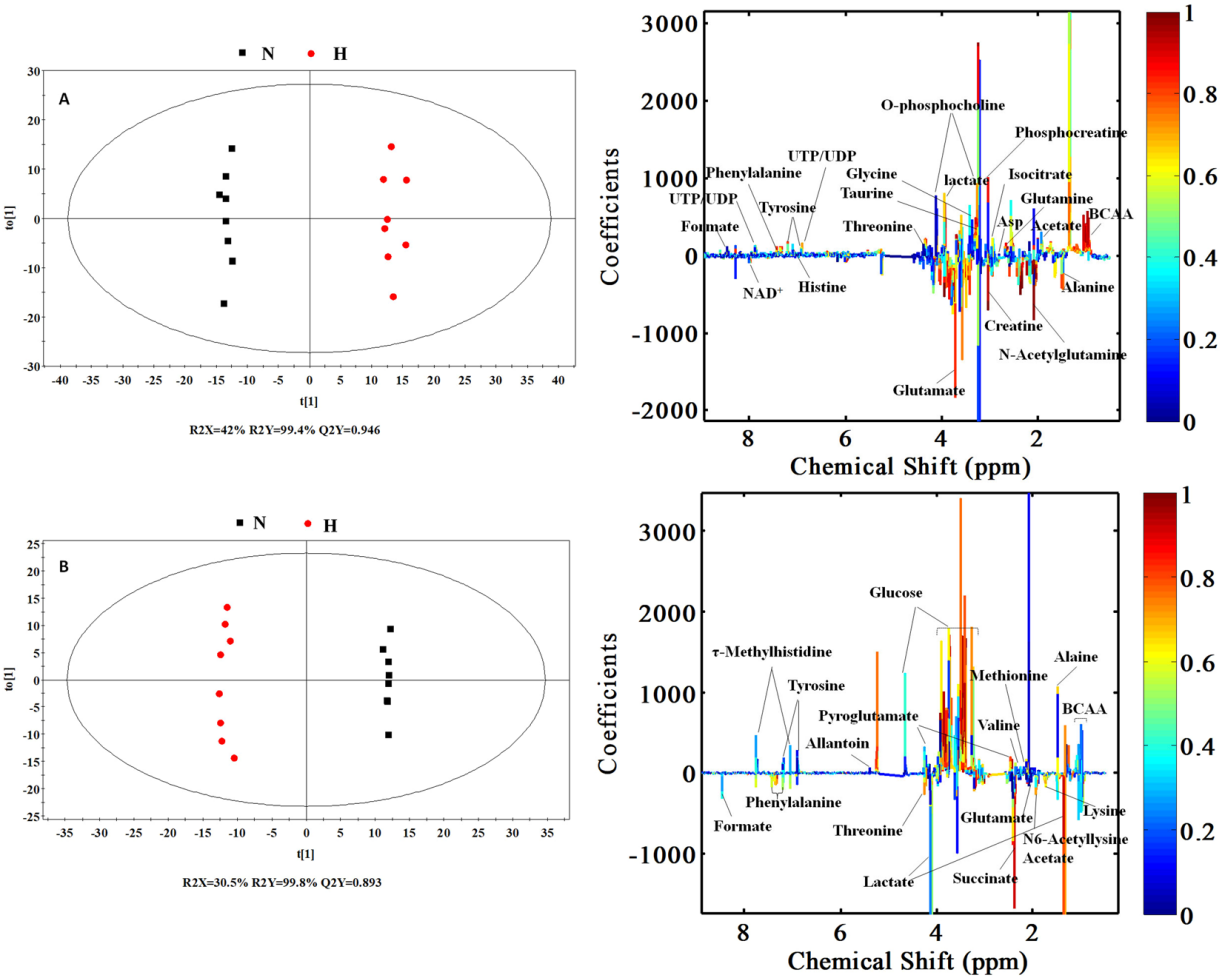
OPLS-DA scores plots (left panel) and corresponding coefficient loading plots (right panel) derived from the ^1^H NMR spectra of cell extracts (**A**) and cell culture media (**B**) samples obtained from different groups. N: normal group; H: high emodin group (100 μM)

An OPLS-DA model was then performed to minimize the possible influence of between-group variability and screen metabonomic differences among the three groups. The significance of the contribution of the identified metabolites to the observed physiological changes was compared. The score plots (Figs 4 and S3) showed distinct separations between the normal group and the emodin exposure groups in the cell extract and culture media samples. A seven-fold cross-validation was applied to estimate the predictive ability of the OPLS-DA models. The parameters for the classification of control versus the high emodin group for the cell extract and cell culture media samples were respectively, R2X = 42%, Q2Y = 0.946 and R2X = 30.5%, Q2Y = 0.993, which demonstrated an acceptable goodness of fit and a high-quality predictability.

### Metabonomic changes and biomarker identification

In the color-coded coefficient loading plots (Fig. 4), valuable biochemical distinctions between the normal group and the high emodin group were identified. The plots depicted considerable metabonomic differences between the control and emodin groups. Multivariate statistical analysis showed increased concentrations of 2-hydroxy-3-methylvalerate, acetate, arginine, aspartate, creatine, isoleucine, leucine, histidine, lactate, lysine, NADP^+^, O-phosphocholine, phenylalanine, taurine, tyrosine, valine and threonine in the cell extracts of the high emodin group and decreased concentrations of 2-hydroxybutyrate, 2-oxoglutarate, alanine, creatine phosphate, glucose, glutathione, glutamine, glycine, isocitrate, N-acetylglutamate, N-acetylglutamine, proline, UDP-glucuronate, ATP, pyroglutamate, N,N-dimethylglycine, betaine, glutamate and N-acetylglycine. The relevant data for the altered metabolites, including chemical shifts, are summarized in Table 1.

**Table 1:** Quantitative comparison of metabolites (33) found in the cell extracts.

Metabolites	Chemical Shift^c^	Integrals in H and N group^a^		Integral in M and N group		Integral in L and N group	
		FC^b^	VIP	FC	VIP	FC	VIP
2-Hydroxybutyrate	**1.64 (m)**	0.8091*	1.2184	0.8772	0.6682	0.8289	1.3138
Acetate	**1.92 (s)**	1.2581**	1.3336	0.9371	0.6518	0.8917	0.9660
Alanine	**1.48** (d), 3.78 (q)	0.7854**	1.4726	0.7198**	1.8466	0.9261	1.0691
Arginine	**1.91 (m)**, 3.24 (t), 3.78 (t)	1.1801**	1.3154	0.9308	0.8094	0.9198	1.0006
Aspartate	**2.68** (m), 3.90 (m)	2.3843**	1.9762	1.3485**	1.7590	1.0918	1.0695
Creatine	**3.04 (m)**	1.4486**	1.6717	1.2119*	1.2233	1.1061	0.9868
Creatine phosphate	**3.05 (s)**, 3.96 (s)	0.7406**	1.9484	0.9241**	1.4916	1.0026	0.1080
Glucose	3.49 (t), 3.72 (dd), **5.24 (d)**	0.5721*	1.1068	0.4758*	1.3593	0.5937*	1.5728
Glutathione	2.97 (dd), **3.77 (m)**	0.4812**	1.9913	0.6360**	2.1425	0.8855	1.8079
Isoleucine	**1.02 (d)**, 1.46 (m)	1.4619**	1.9293	0.9565	0.7286	0.9423	1.0425
Leucine	**0.96 (d)**, 1.69 (m), 3.74 (m)	1.6182**	1.9211	0.9703	0.5433	0.9528	0.8010
Glutamine	2.13 (m), **2.44 (m)**	0.6633**	1.7608	0.8495*	1.3052	0.8998*	1.5323
Glycine	**3.58 (s)**	0.7727**	1.3490	0.7976*	1.3745	0.8868	1.0789
Histidine	**7.09 (s)**	1.6638**	1.6217	1.2003	1.0026	1.0393	0.4060
Isocitrate	2.97 (m), **4.02 (d)**	0.7867**	1.3550	0.7174**	1.7326	0.8080*	1.7588
Lactate	**1.33(d)**, 4.12(q)	1.2566*	1.1083	1.3198**	1.6731	1.2986**	1.9174
Lysine	**1.46 (m)**, 1.73 (m), 3.77 (t)	1.1092*	1.1748	1.1189*	1.2745	1.0932	1.2703
N-Acetylglutamate	2.05 (m), **2.23 (t)**	0.5184**	1.9286	0.5883**	2.1010	0.5746**	2.7253
N-Acetylglutamine	1.93 (dd), **2.12 (s)**	0.5353**	1.9909	0.7079**	2.1119	0.9326	1.3865
NADP+	**8.47(s)**, 8.83(d)	1.4298**	1.3746	1.0824	0.5863	1.0376	0.3253
O-Phosphocholine	3.24 (s), **3.60 (m)**, 4.15 (m)	1.3489**	1.3257	1.0051	0.1356	1.0013	0.2452
Phenylalanine	3.29 (dd), **3.98 (m)**, 7.38 (m)	2.1359**	1.7687	1.3651*	1.3178	1.0465	0.3762
Proline	**3.38 (m)**, 4.15 (m)	0.7138**	1.3935	0.6666**	1.7600	0.7077**	2.2799
Taurine	**3.26 (t)**, 3.42 (t)	1.5803**	1.8060	1.4417**	1.8751	1.1620	1.2841
Tyrosine	**3.06 (dd)**, 3.94 (m), 6.90 (d)	1.2952**	1.7079	1.1039	1.0807	0.9324	0.9615
Valine	**0.99 (d)**, 1.05 (d), 2.28 (m)	1.4327**	1.8617	1.0486	0.7198	0.9751	0.4531
ATP	**4.22(m)**, 4.29(m)	0.7931**	1.3549	0.6575**	1.8749	0.7501**	2.1786
Threonine	**1.33 (d)**, 3.56 (d)	1.2368**	1.5755	1.1438*	1.3897	1.1418	1.4358
N,N-Dimethylglycine	2.92 (s), **3.72 (s)**	0.5694**	1.6912	0.5577**	1.9002	0.7364**	1.9582
Betaine	3.24 (s), **3.89 (s)**	0.7408**	1.6815	0.7418**	1.8939	0.8049**	2.1787
Glutamate	**2.11 (m)**, 2.33(m), 3.75 (m)	0.4799**	2.0016	0.6705**	2.1172	0.8733**	2.1524
2-Oxoglutarate	2.4 (t), **3.01 (t)**	0.8135**	1.4803	0.8325**	1.6518	0.8979*	1.5029
N-Acetylglycine	2.05 (s), **3.74 (d)**	0.7879**	1.3319	0.7194**	1.7719	0.8957	1.0959

^a^The relative integrals of metabolites were determined from the ^1^H NMR analysis of cell extracts of each group. N: normal group; H: high emodin group (100 μΜ); M: middle emodin group (50 μM); L: low emodin group (10 μM).

^b^FC: fold change.

^c^The chemical shifts in bold were used to calculate integrals and p values.

**Compared with the normal group, p < 0.01; *compared with the normal group, p < 0.05.

Metabolite differences in the cell culture media samples were also identified between the emodin-exposure groups and the control group. The levels of 2-methylglutarate, 3-methylglutarate, 4-methylglutarate, allantoin, formate, glutamate, succinate, isoleucine, lactate, N,N-dimethylglycine, N6-acetyllysine, ornithine, pyroglutamate, threonine, valine, τ-methylhistidine and phenylalanine were elevated in the emodin groups, whereas glucose and acetate levels decreased. The altered metabolites are presented in Table 2.

**Table 2:** Quantitative comparison of metabolites (23) found in the cell culture media.

Metabolites	Chemical Shift^c^	Integral in H and N group^a^		Integral in M and N group		Integral in L and N group	
		FC^b^	VIP	FC	VIP	FC	VIP
Alanine	**1.48 (d)**, 3.78 (q)	0.5546**	2.1042	0.7392**	2.1955	0.8930*	1.4488
Allantoin	**5.41 (s)**	1.9970*	1.2710	1.5914	0.6796	1.9288	1.3164
Formate	**8.46 (s)**	1.2739**	2.1095	1.3103**	2.3911	1.2997**	2.6689
Fructose	3.69 (m), **3.82 (m)**, 3.90 (dd)	0.9063**	2.0266	0.9555**	1.9630	1.0569*	1.7715
Glucose	**3.49 (t)**, 3.72 (dd), 5.24 (d)	0.6004**	2.1696	0.7428**	2.3890	0.9310**	1.9516
Arginine	1.91 (m), 3.24 (t), **3.78 (t)**	0.6724**	2.1627	0.8021**	2.3940	0.9427**	1.9785
Methionine	2.14 (s), 2.16 (m), **3.86 (t)**	0.6966**	2.1016	0.8249**	2.1955	0.9507	1.3813
Glutamate	2.11 (m), **2.33 (m)**, 3.75 (m)	1.3120**	1.9648	1.2743**	2.1997	1.1584**	2.3668
Succinate	**2.41 (s)**	2.9409**	2.1890	2.2460**	2.4270	1.1480**	2.3037
Glutamine	2.13 (m), **2.44 (m)**	0.2441**	2.1028	0.3388**	2.3026	0.5654**	2.4428
Lysine	**1.46 (m)**, 1.73 (m), 3.77 (t)	0.7725**	2.0705	0.8596**	2.2625	0.9166**	2.0737
Isoleucine	1.02 (d), **1.46 (m)**	1.2343**	1.9892	1.1539**	1.9826	1.0756*	1.6992
Lactate	1.33 (d), **4.12 (q)**	1.2797**	1.5165	1.1638*	1.3904	1.0022	0.3572
Leucine	0.96 (d), 1.69 (m), **3.74 (m)**	0.6525**	2.1729	0.7808**	2.3874	0.9735	1.0006
N,N-Dimethylglycine	**2.92 (s)**, 3.72 (s)	17.1029**	2.1976	9.1770**	2.4257	3.1164**	2.6144
N6-Acetyllysine	1.41 (m), 1.90 (m), **3.74 (t)**	1.3563**	1.8345	1.2610**	1.8880	1.0904*	1.5103
Ornithine	1.73 (m), **3.05 (t)**, 3.77 (t)	1.6490**	1.9002	1.4071**	1.8105	1.0372	0.5523
Pyroglutamate	2.39 (m), **2.50 (m)**, 4.17 (dd)	1.2075**	1.4501	1.1655*	1.3869	0.9671	0.3876
Threonine	**1.33 (d)**, 3.56 (d)	1.4552**	2.1414	1.3227**	2.3963	1.1463**	2.2457
Tyrosine	3.06 (dd), 3.94 (m), **6.90 (d)**	0.8216**	1.3951	0.8603*	1.3507	1.0823	1.3854
Valine	0.99 (d), 1.05 (d), **2.28 (m)**	1.1046*	1.2260	1.0793**	1.5845	1.0178	0.3643
τ-Methylhistidine	3.24 (dd), 3.70 (s), **7.76 (s)**	3.9045*	1.1948	3.2689*	1.4839	0.6081	0.8213
Phenylalanine	3.29 (dd), 3.98 (m), **7.38 (m)**	1.2297**	1.9856	1.1529**	2.0863	1.0761**	1.8162

^a^The relative integrals of metabolites were determined from the ^1^H NMR analysis of cell culture media for each group. N: normal group; H: high emodin group (100 μΜ); M: middle emodin group (50 μM); L: low emodin group (10 μM).

^b^FC: fold change.

^c^The chemical shifts in bold were used to calculate the integrals and p values.

**Compared with the normal group, p < 0.01; *compared with the normal group, p < 0.05.

The levels of 5 metabolites (threonine, isoleucine, valine, lactate and phenylalanine) were increased in both the cell extract and the cell culture media samples of the emodin-treated groups, and the levels of alanine, glutamine and glucose were reduced. However, the concentrations of glutamate, N,N-dimethylglycine, leucine, arginine and tyrosine in the cell extracts of the emodin treated groups were reduced, while they increased in the cell culture media samples. The opposite trend was observed for the lysine concentration in the cell extract and cell culture media samples.

### Pathway analyses

**Figure 5:**
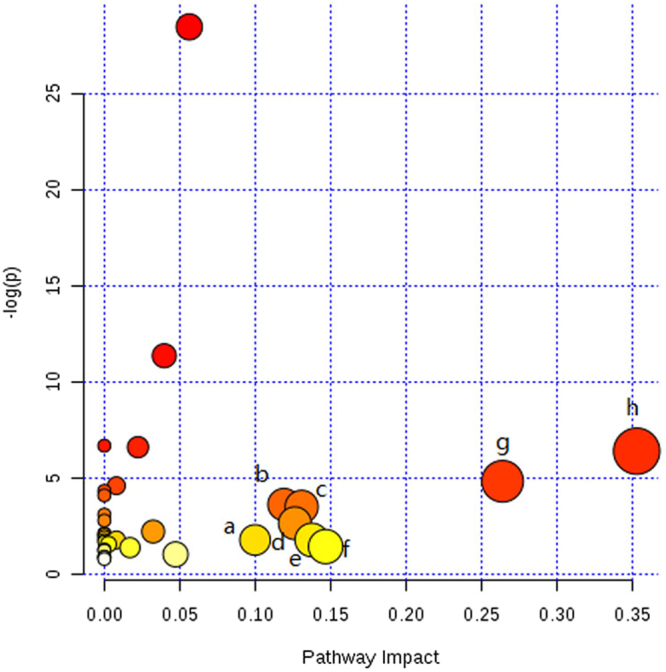
Summary of pathway analysis with MetPA. (**a**) Lysine degradation (**b**) Phenylalanine metabolism (**c**) Glycine, serine and threonine metabolism (**d**) Arginine and proline metabolism (**e**) Pyruvate metabolism (**f**) Lysine biosynthesis (**g**) Alanine, aspartate and glutamate metabolism (**h**) D-Glutamine and D-glutamate metabolism.

Based on the target metabolites, a metabolic pathway analysis was performed using MetPA to reveal the most relevant pathways related to emodin. The potential target pathway was determined using pathway topology analysis and evaluated for an impact value above 0. For this impact value, we found 8 potential target pathways (Lysine degradation; Phenylalanine metabolism; Glycine, serine and threonine metabolism; Arginine and proline metabolism; Pyruvate metabolism; Lysine biosynthesis; Alanine, aspartate and glutamate metabolism; D-Glutamine and D-glutamate metabolism) related to 14 of the metabolites identified in this research. The 8 pathways, which included more than one target, were disrupted across the emodin treatments groups (Fig. 5).

## Discussion

**Figure 6:**
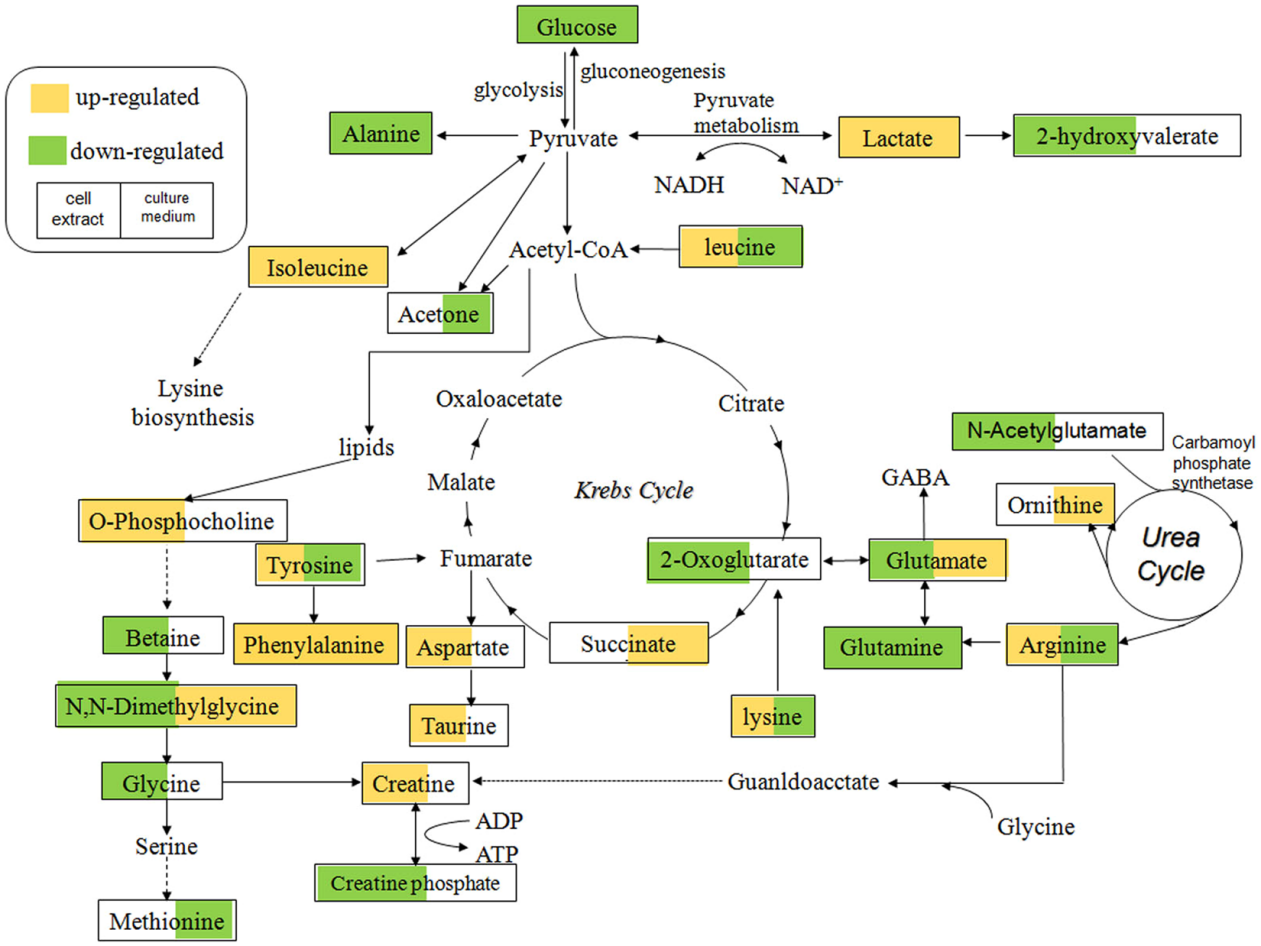
Disrupted metabolic pathways detected by ^1^H NMR analysis. Metabolites in yellow were up-regulated both in the cell extracts and the cell culture media samples; Metabolites in green were down-regulated in both the cell extracts and the cell culture media samples.

Drug-induced hepatotoxicity is a subject of interest for both the toxicology industry and in clinical research. It has been reported that emodin induces P450s, 1A1 and 1B1 in human lung adenocarcinoma CL5 cells^22^. Some recent studies have concluded that emodin suppresses proliferation of HepG2 cells and induces apoptosis^11,23^. Cytotoxicity was also observed in L-02 and BEL cells after exposure to emodin at a concentration of 50 μΜ^24^. There are limited reports on the cytotoxicity of emodin in HepG2 cells^25^. Therefore, we studied the global metabolic therapeutic effects using emodin concentrations of 10, 50 and 100 μM in cultures of HepG2 cells at an experimental exposure time of 24 h. The 100 μM dose of emodin represented the IC50 for cell viability at 24 h of treatment and caused cell apoptosis. We tried to investigate the underlying metabonomic characteristics of emodin in HepG2 cells and the influence of cellular toxicity on small-molecule metabolites. Based on ^1^H NMR, metabonomics were performed to screen for biomarkers of emodin toxicity in HepG2 cells. We demonstrated that the detection of emodin toxicity was possible using metabolic profiling. Finally, 45 compounds concentrations and 8 pathways were disrupted by emodin exposure. Several types of metabolites were detected in the HepG2 cell ^1^H NMR spectra, including some essential amino acids, non-essential amino acids (aspartate, glycine, histidine, arginine, proline, taurine, alanine and glutamate), intermediates of the tricarboxylic acid cycle (TCA) (lactate and isocitrate), ATP and carboxylic acid (2-hydroxyisobutyrate). The culture media NMR spectra were characterized by multiple metabolic intermediates and end-products including the glycolysis and TCA intermediates (lactate and succinate), the metabolic wastes (formate and allantoin) and a series of nutrient substrates, including some amino acids and glucose, which could provide all the essential elements for cell growth. The schematic representation of the metabolic network is shown in Fig. 6.

Metabolites related to energy metabolism. The observed trends in glucose and lactate were opposite in both the cell extract and the cell culture media samples. For example, when glucose decreased, the lactate level increased. Conversely, emodin increased glycolytic activity. Reduced gluconeogenesis and glycolysis promoted acetyl-CoA production for use in the Krebs cycle. However, the acetyl-CoA demand from the Krebs cycle was exceeded by the amount of acetyl-CoA derived from glycolysis, and ketone bodies were thus produced^26^. Accordingly, the level of 2-hydroxybutyrate was reduced in the cell extract in the emodin group. 2-Hydroxybutyrate is a highly energetic compound that transports the energy of liver, and with the help of β-hydroxybutyrate dehydrogenase, it is converted to acetoacetic acid^27,28^. The release of acetate was closely associated with glucose utilization because it is the main method for pyruvate production from glycolysis in the cytoplasm. The level of alanine was closely related to pyruvate and glucose, as it is the primary energy source in the alanine-glucose cycle^29^. As a byproduct of choline metabolism, creatine is formed when guanidinoacetate receives a S-adenosylmethionine (SAM) methyl from guanidinoacetate methyltransferase^30^. Emodin may disrupt energy metabolism and lead to an increase of creatine in HepG2 cells, which was shown to be an emergency energetic regulator^31^.

Metabolites related to the urea cycle and the TCA cycle. Characterized by a decreased utilization of succinate, emodin-induced metabolic changes were determined using the OPLS-DA, which may be related to the low activity of succinate dehydrogenase induced by emodin hepatotoxicity. Arginine, which is produced when arginine-succinate is split, is an important intermediate product in the urea cycle. Catalyzed by arginase in the liver, arginine produces urea and ornithine via hydrolysis, which initiates a new cycle of urea cycle. In the emodin groups, the splitting of arginine-succinate may have been affected by emodin. Arginine release was abnormal and eventually affected the urea cycle and TCA.

Metabolites related to oxidative stress and immune response. As a derivative of glycine, N,N-dimethylglycine is an important intermediate in the metabolism of choline to glycine. Choline is oxidized to betaine, which is then demethylated to form N,N-dimethylglycine. Dimethylglycine is oxidatively demethylated to form sarcosine^32^. It has been reported that N,N-dimethylglycine decreases oxidative stress^33^ and improves immune responses^34^. Compared with the control group, emodin significantly increased the levels of N,N-dimethyglycine and betaine in the cell media samples and decreased the level of N,N-dimethyglycine in the cell extracts. This finding indicates that emodin might influence the biological processes of oxidative stress and immune response.

Metabolism of other amino acids. Due to hepatocellular injury or hepatic ATP consumption, the levels of some amino acids (leucine and Isoleucine) were found to be increased in the HepG2 cell extracts. The concentrations of amino acids in HepG2 cells may vary according to the degree of cell damage. Elevated urinary taurine has long been identified as a specific marker of liver impairments^35–37^, including necrosis and steatosis, which correlate with the hepatocyte necrosis observed by histopathology. Here, the elevated taurine levels in the HepG2 cells may support a disruption of hepatic function. Increased levels of some amino acids including glutamate, threonine, isoleucine, phenylalanine and valine were observed, which suggests that emodin may facilitate the protein catabolism and reduce protein synthesis.

Two pathways (alanine, aspartate and glutamate metabolism and D-glutamine and D-glutamate metabolism) were mainly disrupted by emodin. The results showed a decrease in the levels of alanine, glutamate and glutamine, and an increase in the level of aspartate in the cell extract samples. The level of glutamate increased in the cell culture media samples, while alanine and glutamine decreased. The biosynthesis of these amino acids is connected to intermediates of the citrate cycle. A significant disruption of the citrate cycle was also observed in the emodin exposed cells. Emodin disrupted glutathione metabolism in HepG2 cells, marked by a decreased level of glutathione, which is consistent with results reported by Liu *et al*.^12^. Glutathione is a cellular antioxidant. Therefore, using glutathione to modulate drug metabolism is an important mechanism for drug detoxification^13^. As a substrate, aspartate can be used to produce glutamate and oxaloacetic acid, and glutamate is one of the substrates used to synthesize glutathione. With the depletion of glutathione, the toxicity of emodin is increased, resulting in oxidative stress and peroxidation reactions.

The liver is an essential organ for drug and xenobiotic metabolism. Drug-induced hepatotoxicity may result from the direct toxicity of compound or from indirect toxicity due to its active metabolites. One limitation of our study is that we were only able to detect and conduct qualitative analysis on metabolites that were disrupted by emodin exposure in HepG2 cells. We were thus not able to directly measure the pathways and mechanisms affected. Future studies should identify accurate biomarkers, the mechanism of action for emodin and how to intervene to reduce emodin’s toxic effects.

## Conclusions

In this study, we explored emodin toxicity using a combination of NMR and pattern recognition data to establish cell metabonomics from samples of cell extracts and cell culture media. Emodin disrupted several classes of metabolites and disrupted biological processes including the Krebs cycle, amino acid metabolism and purine metabolism. Moreover, this study may contribute to the development of new strategies to elucidate TCM toxicity information, identify potential targets for candidate drugs and clarify the relevant signal network.

## Materials and Methods

### Cell culture and viability

The HepG2 cells were purchased from the Shanghai Institutes for Biological Sciences, Chinese Academy of Sciences (Shanghai, China) and used within twenty passages. HepG2 cells were maintained in high-glucose Dulbecco’s-modified eagle medium (DMEM) with 10% fetal calf serum. Cells were cultured in a humidified atmosphere at 37 °C and with 5% CO_2_.

Cell viability was evaluated using an MTT assay^38^. HepG2 cells were seeded in a 96-well microtiter plate with 1 × 10^4^ cells/well left to adhere overnight before treatment. Emodin (purchased from the National Institute for Food and Drug Control) was diluted in dimethylsulfoxide (DMSO) and high-glucose DMEM and added to the cells for 12 h or 24 h incubations. The final concentration was 0.78 to 200 μM. The cell media, which contained only DMSO, was used as a control. Absorbance was determined at 570 nm using an ELISA reader. Three replicates were performed for each cell group.

### DAPI staining

HepG2 cells (approximately 1 × 10^6^ cells/well) were seeded in 6-well plates. Three different concentrations of emodin (10, 50, 100 μM) were added to the adherent cells and incubated for 24 h. At room temperature, HepG2 cells were stained by DAPI (5 μg/mL) for 10 min. The DAPI dye was then rinsed out. The changes in the cells were observed and the stained cells were photographed.

### Sample preparation

There were four study groups: a high emodin group (100 μM, HG), a middle emodin group (50 μM, MG), a low emodin group (10 μM, LG) and control group (normal cultured HepG2 cells). Approximately 1 × 10^6^ cells/well of HepG2 cells were seeded in 6-well plates and left to adhere overnight. Three different concentrations of emodin (10, 50, 100 μM) were then added to the cells and incubated for 24 h.

Cell extract preparation: Cell extracts were prepared using a precooled methanol-chloroform-water system (2:2:3). After removing the culture media from the culture dish, cells were quickly washed three times with ice-cold PBS (pH 7.4) to remove media components. Cells were then quenched with 1.4 ml of methanol and 0.7 ml of ultrapure H_2_O and homogenized. Next, 1.4 ml of chloroform and 1.4 ml of ultrapure H_2_O were added to the homogenate and mixed for extraction. The solution was centrifuged at 10,000 g for 15 min at 4 °C. The aqueous phase was lyophilized for the next metabonomic analysis. Lyophilized cell extracts were resuspended with D_2_O and a sodium phosphate buffer. Samples were mixed uniformly and centrifuged at 10,000 g for 10 min at 4 °C. The supernatant was collected for further assessment.

Cell culture media preparation: culture medium samples were prepared using 200 μL of cell media mixed with 400 μL of D_2_O^19^. All samples were mixed and then centrifuged at 10,000 g for 10 min at 4 °C. The supernatants were collected for further assessment.

### ^1^H NMR spectroscopy

All samples were analyzed by ^1^H NMR spectroscopy using a VARIAN VNMRS 600 MHz NMR spectrometer (Varian Inc, Palo Alto, Calif) operating at 298 K and 599.871 MHz using a 5-mm inverse-proton triple resonance probe. For intracellular metabolite extracts, the standard NOESYPR ^1^D pulse sequence (RD–90°–t1–90°–tm–90°–ACQ) was used with the irradiations at the water frequency during a recycle delay of 2 s and a mixing time of 100 ms to suppress the residual HOD signal.

The ^1^H NMR spectra of the cell culture media were obtained by the water-suppressed standard 1D Carr-Purcell-Meiboom-Gill pulse sequence (RD-90°-(τ−180°-τ) n-ACQ). The free induction decays (FIDs) were recorded by 64 K data points with a spectral width of 12000 Hz and 128 scans with a relaxation delay of 2.0 s and an acquisition time of 1.36 s. The FIDs were weighted by an exponential function with a 0.5 Hz line-broadening factor prior to Fourier transformation.

### Data processing

The ^1^H NMR spectra were processed using MestReNova 7.1.2 software (Mestrelab Research, Spain). All ^1^H-NMR spectra were manually corrected for baseline and phase and referenced to the TSP signal (δ0.00) to assess the changes in the endogenous metabolites related to the emodin toxicity. In the cell extract NMR spectra, the regions of δ 5.2–4.6 and δ2.8-2.69 were removed to eliminate the water suppression and DMSO signals. For the NMR spectra of the media, the regions of δ5.19-4.68 and δ3.37-3.33, δ2.89-2.58 and δ3.69-3.61, δ1.23-1.15 were excluded to eliminate the effect of residual water, methanol, DMSO and ethanol signals, respectively.

Multivariate analysis was performed with SIMCA-P + 12.0 (Umetrics, Sweden). Principal component analysis (PCA) was applied to mean-centered data to identify outliers and to acquire the data distribution profiles. To improve the separation caused by variations among the groups and minimize other biological analytical variations, sample classes were modeled using the orthogonal partial least squares discriminant analysis (OPLS-DA) algorithm with a unit variance-scaled approach. Coefficient plots were generated with MATLAB scripts (http://www.mathworks.com) with some in-house modifications and color-coded by the absolute value of coefficients^39^.

To determine which variables contributed to the assignment of spectra among the groups, we analyzed the variable importance in the projection (VIP) values of each peak from the OPLS-DA models in this study. To detect significant differences in the signals between the two groups, an independent T-test was conducted in SPSS Statistics 17.0 (SPSS Inc, USA). Differences in metabolites were identified as having statistically significant results for both the multivariate analyses with VIP > 1 and the univariate analyses with p < 0.05. Additionally, the significance of the metabolites was assessed based on the unpaired Student’s T-test of chemical shifts. Most of the metabolites were identified by comparing them with the HMDB (http://www.hmdb.ca/) and the previous literature, which will be listed in the results and references.

### Ethical approval

All applicable international, national, and/or institutional guidelines for the care and use of animals were followed.

## Electronic supplementary material

Supplementary Material
