# Supplementary material for: NMR-based Metabolomic Techniques Identify the Toxicity of Emodin in HepG2 Cells
Source: Sci Rep. 2018 Jun 20;8:9379. doi: 10.1038/s41598-018-27359-4 (PMC6010407; doi:10.1038/s41598-018-27359-4)
Supplement: Supplementary file 1 — Supplementary Material [file 41598_2018_27359_MOESM1_ESM.doc]

**NMR-based Metabolomic Techniques Identify the Toxicity of Emodin on HepG2 Cells**

**Chang Chen^1†^, Jian Gao^1 ,2†^, Tie-Shan Wang^2^, Cong Guo^1^, Yu-Jing Yan^3^, Chao-Yi Mao^1^, Li-Wei Gu^1^, Yang Yang^4^****, Zhong-Feng Li^3^* & An Liu^1^***

**^†^** *Chang Chen and Jian Gao contributed equally to this work.*

*^1^ Institute of Chinese Materia Medica, China Academy of Chinese Medical Sciences, Beijing, China ,^2^Beijing University of Chinese Medicine, Beijing, China, ^3^ Department of Chemistry, Capital Normal University, Beijing, China, ^4^ China Academy of Chinese Medical Sciences, Beijing, China*

*Correspondence should be addressed to An Liu: aliu@icmm.ac.cn and Zhong-Feng Li: [lizf@cnu.edu.cn](mailto:lizf@cnu.edu.cn)


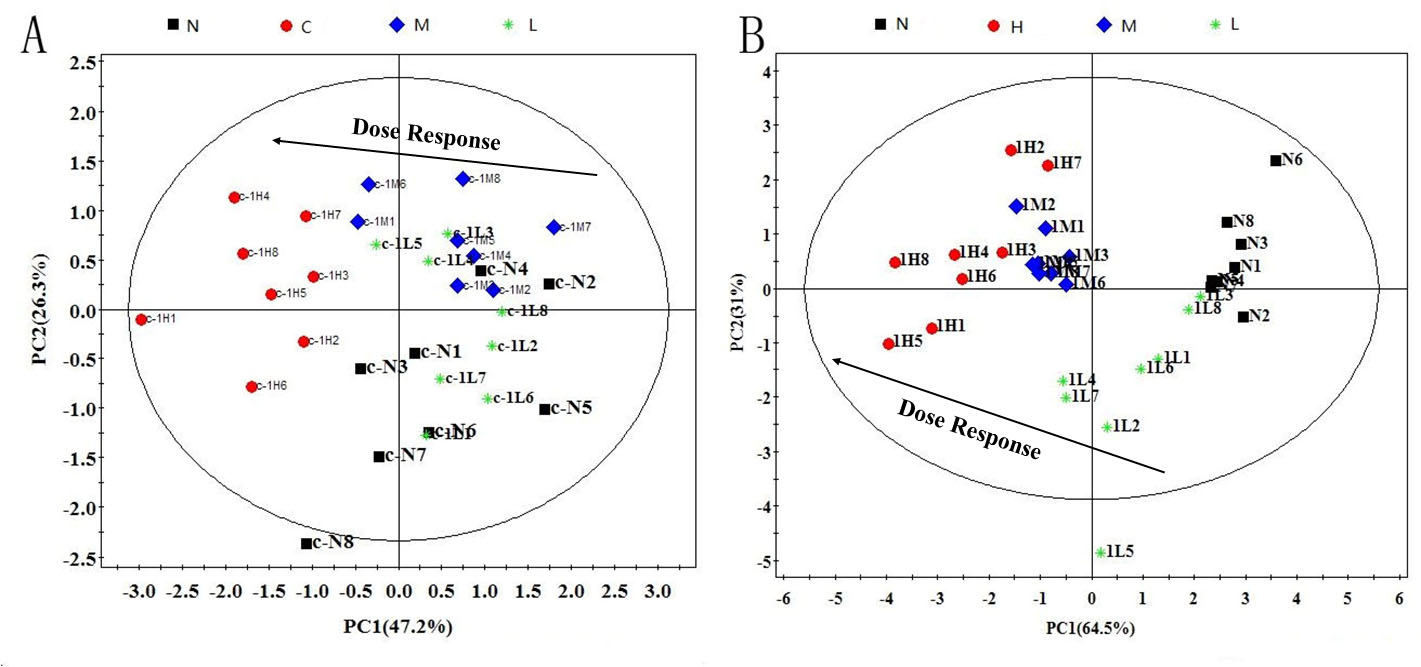


**Fig.S1** PCA scores plot for cell extracts (A) and cell culture media (B). N: normal group; H: high emodin group (100 μΜ); M: middle emodin group (50 μM); L: low emodin group (10 μM).


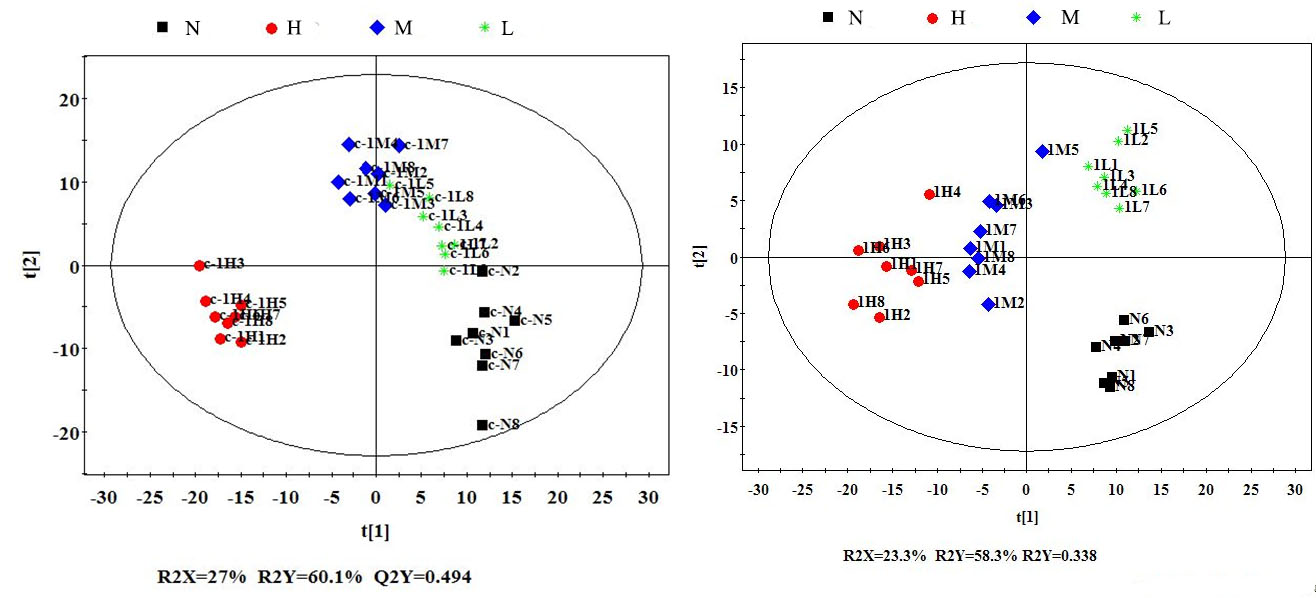


**Fig.S2** PLS-DA scores plot for cell extracts (A) and cell culture media (B). N: normal group; H: high emodin group (100 μΜ); M: middle emodin group (50 μM); L: low emodin group (10 μM).


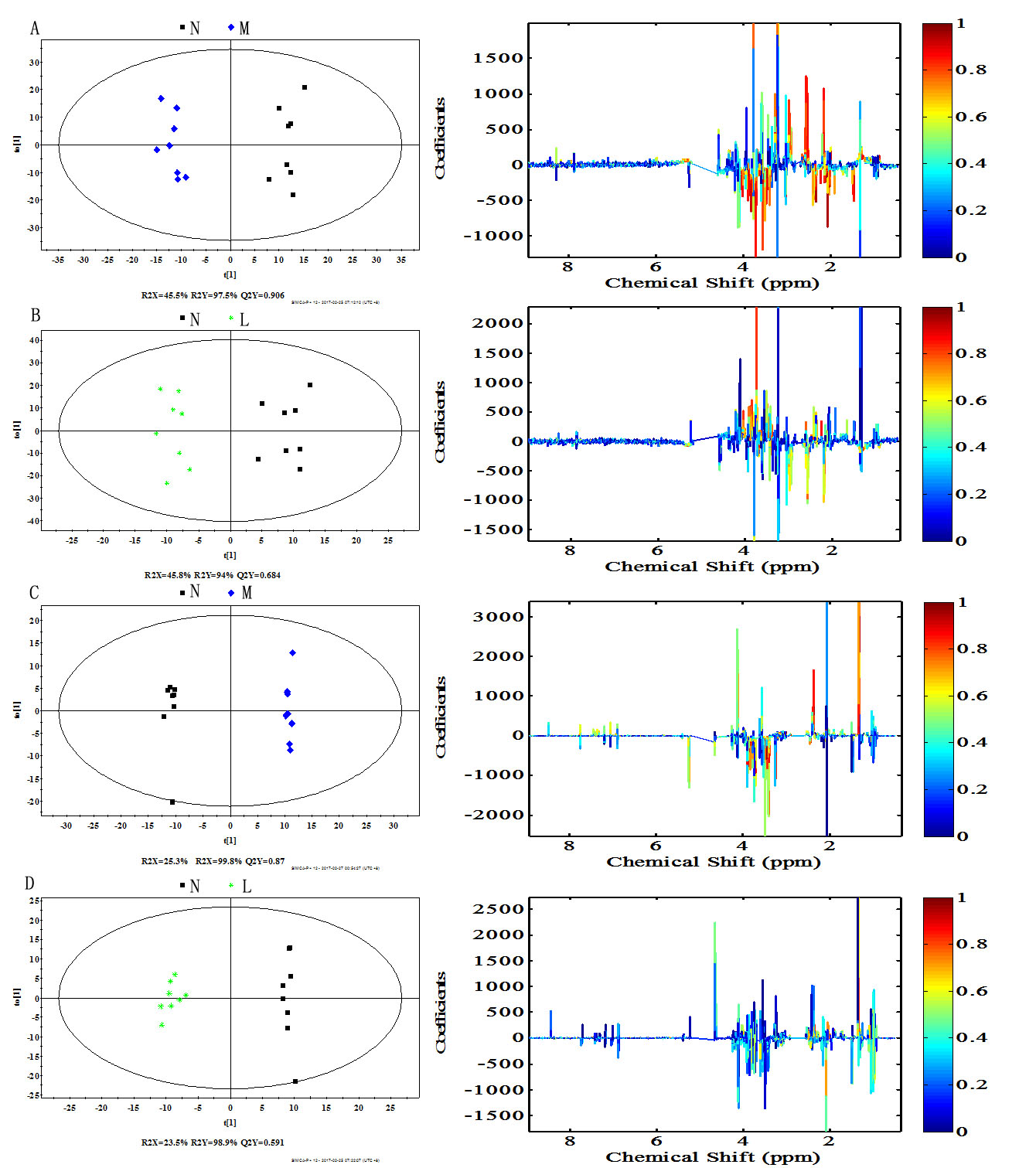


**Fig.S3** OPLS-DA scores plots (left panel) and corresponding coefficient loading plots (right panel) derived from ^1^H NMR spectra of cell extracts (A) and cell culture media (B) obtained from different groups.

N: normal group; M: middle emodin dosage group (50 μM); L: high emodin dosage group (10 μM)
